# Mobile Intervention to Address Cannabis Use Disorder Among Black Adults: Protocol for a Randomized Controlled Trial

**DOI:** 10.2196/52776

**Published:** 2024-02-19

**Authors:** Pamella Nizio, Bryce Clausen, Michael S Businelle, Natalia Ponton, Ava A Jones, Brooke Y Redmond, Julia D Buckner, Ezemenari M Obasi, Michael J Zvolensky, Lorra Garey

**Affiliations:** 1 University of Houston Houston, TX United States; 2 TSET Health Promotion Research Center Stephenson Cancer Center University of Oklahoma Health Sciences Center Oklahoma City, OK United States; 3 Louisiana State University Baton Rouge, TX United States

**Keywords:** cannabis use, false safety behaviors, mobile health, just-in-time adaptive interventions, Black or African American, mobile phone, African American, Black, cannabis, adults, adult, Hispanic, Latin, adaptive intervention, cannabis reduction, cessation, ecological momentary assessments

## Abstract

**Background:**

African American or Black (hereafter referred to as Black) adults who use cannabis use it more frequently and are more likely to meet criteria for cannabis use disorder (CUD) than both White and Hispanic or Latin individuals. Black adults may be more apt to use cannabis to cope with distress, which constitutes a false safety behavior (FSB; a behavior designed to reduce psychological distress in the short term). Although FSB engagement can perpetuate the cycle of high rates of CUD among Black individuals, limited work has applied an FSB elimination treatment approach to Black adults with CUD, and no previous work has evaluated FSB reduction or elimination in the context of a culturally tailored and highly accessible treatment developed for Black individuals.

**Objective:**

This study aims to develop and pilot-test a culturally tailored adaptive intervention that integrates FSB reduction or elimination skills for cannabis reduction or cessation among Black adults with probable CUD (Culturally Tailored-Mobile Integrated Cannabis and Anxiety Reduction Treatment [CT-MICART]).

**Methods:**

Black adults with probable CUD (N=50) will complete a web-based screener, enrollment call, baseline assessment, 3 daily ecological momentary assessments (EMAs) for 6 weeks, and a follow-up self-report assessment and qualitative interview at 6 weeks after randomization. Participants will be randomized into 1 out of the 2 conditions after baseline assessment: (1) CT-MICART+EMAs for 6 weeks or (2) EMAs only for 6 weeks.

**Results:**

The enrollment started in June 2023 and ended in November 2023. Data analysis will be completed in March 2024.

**Conclusions:**

No culturally tailored, evidence-based treatment currently caters to the specific needs of Black individuals with CUD. This study will lay the foundation for a new approach to CUD treatment among Black adults that is easily accessible and has the potential to overcome barriers to treatment and reduce practitioner burden in order to support Black individuals who use cannabis with probable CUD.

**Trial Registration:**

ClinicalTrials.gov NCT05566730; https://clinicaltrials.gov/study/NCT05566730

**International Registered Report Identifier (IRRID):**

DERR1-10.2196/52776

## Introduction

Cannabis has been among the most widely used substances for 30 consecutive years in the United States [1], and rates of past-year use have consistently increased in the general population [2,3]. Among individuals who use cannabis, African American or Black (hereafter referred to as Black) individuals exhibit more severe use patterns, including weekly use, and are more likely to meet diagnostic criteria for current cannabis use disorder (CUD) than both White and Hispanic or Latin individuals who use cannabis [4-6]. These data are alarming as CUD is associated with more severe psychosocial risk profiles relative to cannabis users without CUD and nonusers, including poly-substance use, psychiatric problems, and legal trouble [7]. Additionally, although Black individuals who use cannabis are more likely to report being ready to quit and making a recent quit attempt than both Hispanic or Latin and White individuals who use cannabis [8,9], this population is less likely to seek in-person treatment relative to White individuals who use cannabis. Specific individual (eg, beliefs about use) [10], community (eg, neighborhood attitudes about use) [11], and institutional (eg, health care access) [12] factors, as well as institutionalized racism and discrimination (eg, more likely to not be listened to by practitioners) [13], likely contribute to the reluctancy to seek traditional treatments among Black individuals with CUD. Given that treatment involvement has been shown to assist with cannabis use reduction and prolonged abstinence [14], addressing the lack of treatment engagement among this population is imperative to reduce the potential negative health and psychological effects of cannabis use among this group.

Psycho-sociocultural models of substance use posit that Black individuals may use cannabis and continue using it despite cannabis-related problems to manage psychological distress associated with minority-related stress and daily stressors [15-18]. Using cannabis to cope with such distress reflects a false safety behavior (FSB), or a behavior designed to reduce psychological distress in the short term but paradoxically maintains or even exacerbates distress in the long term [19,20]. FSB is more frequent among Black individuals compared to non-Hispanic or Latin White individuals and it is associated with more anxiety, depression, and suicidal thoughts and behaviors among Black individuals [21]. Thus, FSB is an important behavioral vulnerability factor associated with mental health problems among Black individuals. These findings are concerning as problematic cannabis use is higher among those with mental health problems [22], and mental health problems interfere with changing cannabis use [23]. Moreover, the majority of individuals who use cannabis tend to engage in additional FSBs such as avoidance and avoiding social situations when cannabis is unavailable [24], and FSBs may contribute to cannabis-related problems due to tendencies to engage in maladaptive attempts to regulate negative affect [25]. As such, FSB engagement can perpetuate the cycle of high rates of CUD among Black individuals who use cannabis and increase the risk for poor psychosocial outcomes and cannabis-related disparities [24,26,27].

Importantly, transdiagnostic treatments have been developed to eliminate FSBs to improve behavioral health outcomes. Specifically, cognitive-behavioral approaches to FSB elimination treatment that target the identification and elimination of FSBs have been designed and successfully used for use across various anxiety disorders [20]. Furthermore, recent work has explored the potential of FSB elimination integrated with motivation enhancement therapy combined with cognitive behavioral therapy (MET-CBT) to address the co-occurrence among anxiety and CUDs, Integrated Cannabis and Anxiety Reduction Treatment (ICART) [28-30]. In a pilot test of ICART, individuals with CUD were randomized to either in-person ICART or MET-CBT for CUD [28]. Although participants in both study groups reported decreased cannabis use and related problems, patients in the ICART condition were more likely to be abstinent posttreatment than those in the MET-CBT condition. Patients with more severe baseline cannabis use and use-related problems were especially likely to benefit from ICART [31]. These findings suggest that integrating FSB elimination with MET-CBT is at least as efficacious, if not more efficacious, as a gold-standard psychosocial CUD treatment (MET-CBT), especially for patients with more severe cannabis-related pathology.

Despite the potential for FSB reduction or elimination treatments to assist with cannabis use reduction or cessation among individuals with problematic use, this work has been limited by its in-person design and lack of cultural tailoring to Black adults. Indeed, given the cannabis-related and FSB disparities experienced by Black adults [16,21], it may be beneficial to evaluate FSB reduction or elimination integrated with cognitive behavioral therapy (CBT) for CUD in the context of a culturally tailored and highly accessible treatment (ie, a mobile health [mHealth] intervention delivered via a smartphone app) developed for this underserved group.

Extant literature has highlighted existing mHealth work for cannabis use and CUD [32], including mHealth interventions that have been culturally tailored to treat specific populations such as individuals with psychosis [33], as well as individuals with comorbid CUD and cigarette smoking [34]. However, no mHealth interventions for cannabis use and CUD currently target specific psycho-sociocultural factors related to cannabis use and use-related problems among Black individuals who use cannabis with probable CUD. We have therefore developed and are currently pilot-testing a culturally tailored intervention for Black adults with probable CUD through integrated FSB reduction or elimination with CBT for CUD (Culturally Tailored-Mobile Integrated Cannabis and Anxiety Reduction Treatment [CT-MICART]) using an accessible, adaptable, and highly scalable smartphone app. The goal is to examine the effects of CT-MICART on cannabis use, coping motives for cannabis use, and FSB engagement. We hypothesize that participants who are randomized into the CT-MICART condition will report better cannabis outcomes and less FSB compared to those who are randomized into the control condition at the 6-week follow-up. Furthermore, we will examine app engagement indicators and review qualitative data for methods to improve app content.

## Methods

### Ethical Considerations

All participants provided written informed consent signed electronically after reviewing consent documents with research staff. To protect participant privacy and confidentiality, all phone and Zoom (Zoom Video Communications) appointments are completed by trained research staff in a secure office. Additionally, participants are assigned an ID number that is used to identify their data throughout the study. Only trained research staff have access to the key that can match the participant data to the participant’s name. The key is password-protected on a secure server housed at the University of Houston. Participants are compensated up to US $160 in Amazon electronic gift cards for participating in the study. The institutional review board (IRB) at the University of Houston approved the study (STUDY00003690).

### Study Design

**Figure 1:**
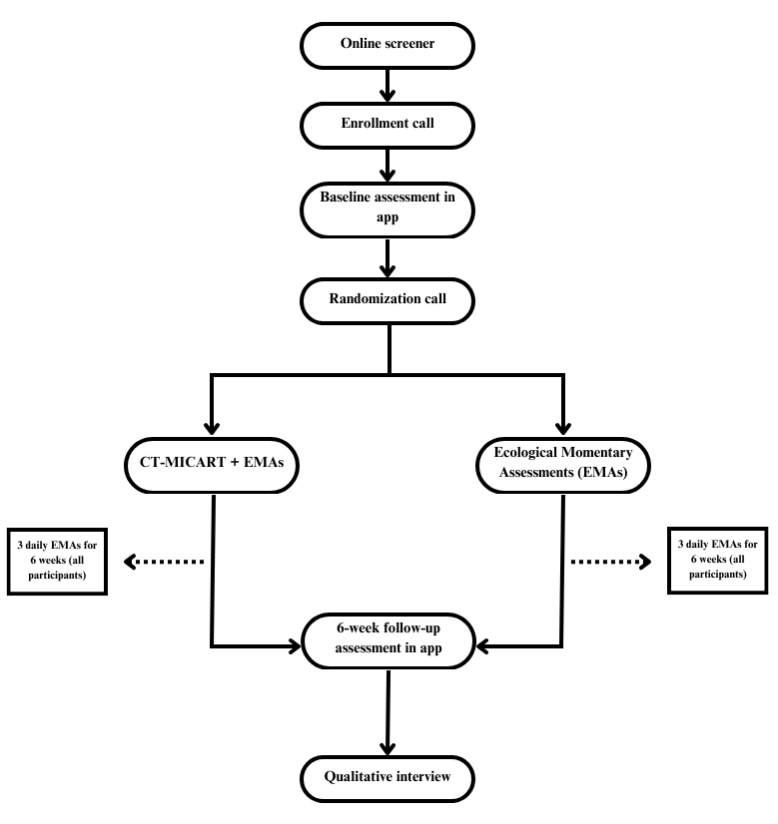
Study flowchart. CT-MICART: Culturally Tailored-Mobile Integrated Cannabis and Anxiety Reduction Treatment; EMA: ecological momentary assessment.

A total of 50 Black adults with probable CUD are being recruited through national advertisements across different social media and web-based platforms to participate in this study. Eligible participants are randomized into 1 out of the 2 conditions (ecological momentary assessment [EMA] only vs CT-MICART+EMA). All the participants who consented are asked to complete 3 prompted daily EMAs for 6 weeks. Only participants in the CT-MICART+EMA condition will receive intervention content. Participants are informed during the consent process that they have an equal chance of being assigned to each study condition. Moreover, participants are also informed that one of the study’s treatment conditions consists of watching treatment videos and using CT-MICART app features, while the other condition consists only of completing daily EMAs. Following the 6-week intervention period, all participants will complete a follow-up assessment survey in the app and a qualitative interview phone call. See Figure 1 for the study flow.

### Participants

Participants will include 50 individuals who identify as Black with probable CUD. Participants must meet the following eligibility criteria to participate: (1) at least 18 years of age, (2) self-identify as Black or African American, (3) meet criteria for probable CUD (assessed via the Cannabis Use Disorder Identification Test-Revised [CUDIT-R] with a score of >12) [35], (4) motivated to reduce cannabis (>5 on a 10-point scale), (5) score ≥4 on the Rapid Estimate of Adult Literacy in Medicine-Short Form (REALM-SF) indicating higher than sixth-grade English reading level [36], (6) own an Android smartphone for EMA completion, and (7) report cannabis use to manage anxiety or stress in the past month. Exclusion criteria include (1) legal mandate of substance misuse treatment; (2) report of current or intended participation in a concurrent substance use treatment, including pharmacotherapy or psychotherapy for CUD not provided by the researchers; (3) ongoing psychotherapy of any duration directed specifically toward the treatment of anxiety or depression; (4) not being fluent in English; (5) pregnant or planning to become pregnant within the next 6 months (assessed via self-report); and (6) inability to provide a photo ID and valid address to verify identity.

### Procedures

This study is funded by the National Institute on Minority Health and Health Disparities (U54MD015946) and is registered on ClinicalTrials.gov (NCT05566730). The IRB where the study takes place has reviewed and approved all procedures and study materials. Interested participants are asked to complete a web-based self-report screener survey via Qualtrics (Qualtrics International Inc). Those deemed eligible at the screener are then scheduled for an enrollment Zoom call of approximately 30 minutes. During the enrollment Zoom call, participants are given detailed information on the goals, purpose, and procedures of the study; provide informed consent; show their photo ID to the research team; and complete a literacy test to confirm they are at higher than sixth-grade English reading level [37]. Those found eligible during the enrollment Zoom call are asked to download the Insight smartphone app onto their personal smartphone and to complete the 30-minute baseline survey via the app. Eligible participants who complete the baseline survey are then contacted by research staff so they can be randomized into a study condition (ie, CT-MICART vs EMA only). During this randomization call, participants are oriented to app features for their assigned condition by the research team.

**Figure 2:**
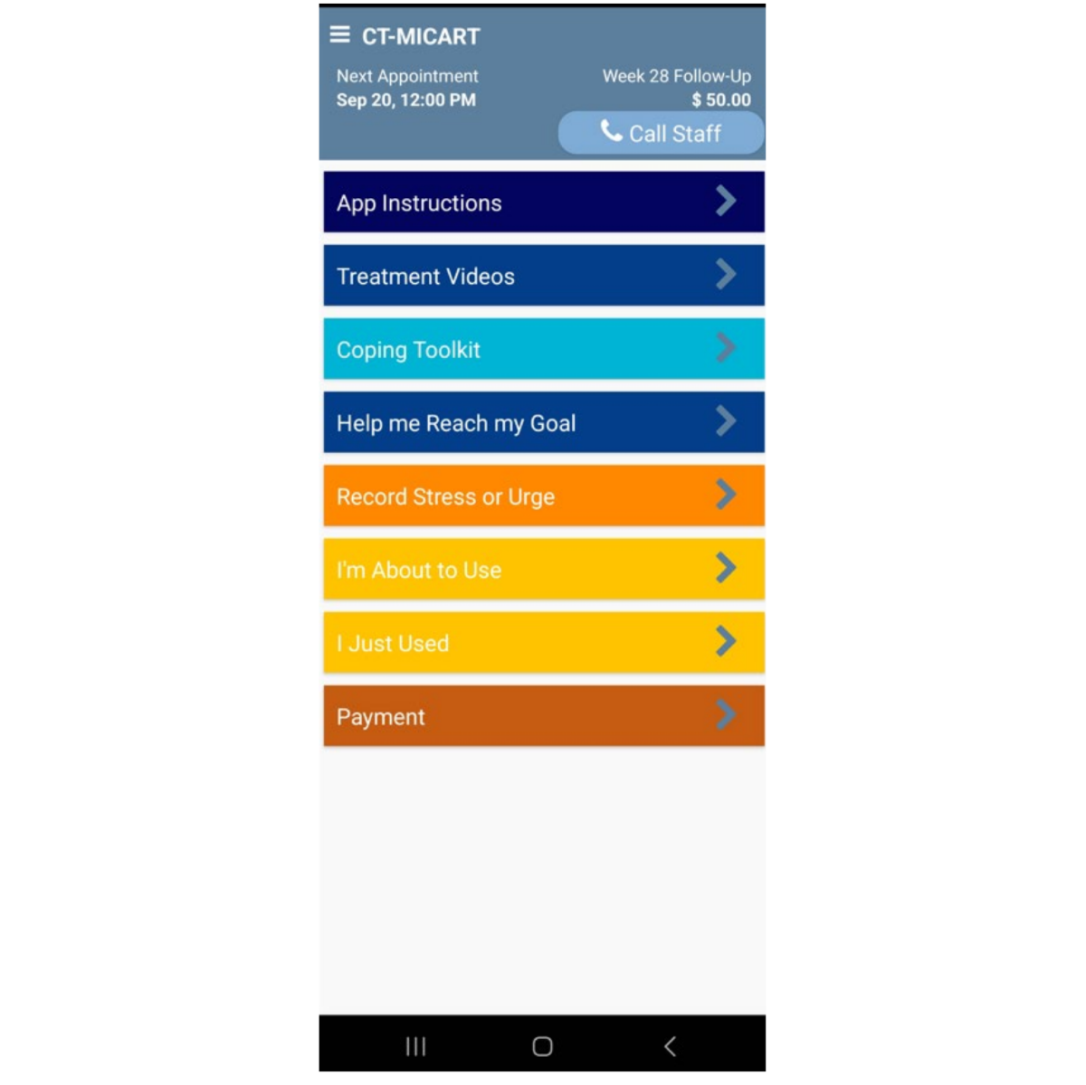
The main menu of CT-MICART features. CT-MICART: Culturally Tailored-Mobile Integrated Cannabis and Anxiety Reduction Treatment.

Following randomization, participants are prompted by the app to complete 3 EMAs daily. EMAs take approximately 2-3 minutes to complete. The research staff monitors the EMA completion rates of each participant on a weekly basis. When a participant’s completion rate falls below 80%, a research staff member contacts the participant via text, phone, or email to remind them of the importance of completing their daily EMAs. Participants are informed that they can contact study staff at any point during the study by pressing the “Call Staff” button at the top of the app screen should they experience technical difficulties. Please see Figure 2 for a screenshot of the CT-MICART features available via the app home screen.

All participants are also asked to complete a 6-week postrandomization assessment and a qualitative interview. Specifically, at the end of the 6-week EMA period, the follow-up assessment becomes available on the app home screen and takes approximately 30 minutes to complete. The qualitative interview is scheduled with a trained research staff member and focuses on the participant’s experiences using the CT-MICART+EMA or EMA-only app features. Participants can earn up to US $160 in Amazon electronic gift cards for their participation. Specifically, eligible participants are compensated with a US $20 gift card for completing the baseline assessment and a US $50 gift card for completing the 6-week follow-up, including the qualitative interview. Ineligible participants are not being compensated for completing the brief baseline Zoom call. In addition to baseline and follow-up compensation, participants are compensated for their EMA completion based on their completion rate. Those who complete 50% to 70% of all EMA assessments during the 6-week study period will receive a US $30 gift card, those who complete 71% to 79% will receive a US $60 gift card, and those who complete 80% or more will receive a US $90 gift card. Thus, if a participant completes 80% or more of the assessment across the entire 6-week trial, they can earn up to US $160 in electronic gift cards across all assessments.

### Intervention Conditions

#### Overview

All participants have access to a “Call Staff” button that enables participants to easily call the study team. In addition, all participants have access to an “App Instructions” button that provides detailed descriptions of each of the app functions. Finally, all participants are informed that they can click the “Payment” button to view an up-to-the-moment accounting of all EMAs prompted and completed and the current compensation based on EMA and assessment completion.

All participants are instructed to complete all smartphone assessments through Insight (TSET Health Promotion Research Center), an encrypted mobile app through which participants receive all study content. Encrypted data will be automatically password protected and saved to the University of Houston’s and the University of Oklahoma Health Science Center’s institutional server and will only be accessible to the IRB-approved research team members. Moreover, participants are informed during the consent process that they are responsible for any phone service costs related to the study.

#### Active Condition (CT-MICART+EMAs)

##### Overview

The CT-MICART+EMA condition consists of access to the CT-MICART content in the Insight app and 3 prompted daily EMAs. The morning EMA is delivered 30 minutes after the preset waking time, the lunch EMA is delivered at 12:15 PM, and the evening EMA is delivered 1 hour and 15 minutes before the participant’s preset sleep time. The core components of the CT-MICART condition include FSB elimination training and CBT for CUD with culturally tailored content. The CT-MICART+EMA content includes (1) treatment on a “schedule” that is culturally adapted (ie, 12 different 3- to 5-minute treatment video files); (2) participant-driven, automated, individually tailored treatment messages (eg, tailored to each participant’s daily goal of reducing cannabis use, abstaining from cannabis, or no daily goal); (3) “on demand” features (ie, Coping Toolkit and Help Me Reach My Goal); and (4) end-of-day FSB elimination training exercises.

##### Process to Culturally Adapt Treatment

We followed the Cultural Adaptation Process [38] model of treatment adaption to culturally tailor CT-MICART. All treatment videos include depictions of Black adults and accompanying audio is voiced by Black voice actors who worked closely with our research team. Moreover, all app content was reviewed by a diverse Community Research Advisory Board (CRAB) at the Health Research Institute at the University of Houston. The CRAB was consulted: they provided feedback during the app development process, and appropriate modifications were made based on their feedback.

##### Treatment on a Schedule

To provide automated intervention content that is tailored to each participant’s current goals and delivered in a manner that is best matched to their schedule, the app asks participants if they would like to watch 1 of the 12 different 3- to 5-minute treatment videos (ie, 2 videos per week) over the 6-week intervention period. Scheduled video sessions are cued (ie, the phone rings and vibrates) at the scheduled time and every 15 minutes (up to 2 times) after the scheduled time until the participant acknowledges the cue. Participants have the option to delay or reschedule videos or watch them after the scheduled day or time (ie, the next unwatched video populates [in order] in the app after the previous video is viewed). Videos can be watched as many times as desired. The phone records the date or time when each video is watched (ie, both initiation and completion). The 12 brief (3-5 minutes) CT-MICART videos provide psychoeducation on (1) the nature of cannabis use and negative affect (eg, anxiety and stress) and the FSB model; (2) the emotional processing model of negative affect (and how it relates to cannabis) and cannabis-related coping strategies (eg, avoiding people, places, and things); (3) relations between FSB and racial discrimination, negative affect, and cannabis use; (4) cannabis (and other substance) use as an FSB and understanding cannabis use patterns; (5) countering phobias (ie, exposure to anxiety- and stress-provoking stimuli without engagement in FSB, including cannabis) and coping with cravings; (6) fading other FSBs (eg, checking, reassurance seeking, companions, and avoidance of bodily sensations) and managing thoughts related to cannabis use; (7) fading other FSBs (avoidance) and managing negative moods; (8) fading other FSBs (cognitive avoidance) and seemingly irrelevant decisions; (9) problem-solving; (10) managing social influences (refusal skills and assertiveness); (11) planning for emergencies and coping with lapse; and (12) preventing relapse.

##### Tailored and Real-Time Treatment Messages

Participants receive personally tailored messages that are based upon each day’s cannabis cessation or reduction goal (ie, reduce cannabis use today, abstinent from cannabis today, or no cannabis use goal today). Thus, the app “meets participants where they are” and provides messages that is in line with their current goal. On days when the participant has no goal to reduce or abstain from cannabis, the app offers primarily “gain-framed” messages that aim to increase motivation for cannabis cessation or reduction. Importantly, the type of message that is delivered at the end of each EMA is recorded in the database so we may analyze the effect of messages on currently present cannabis use triggers.

##### On-Demand Features

**Figure 3:**
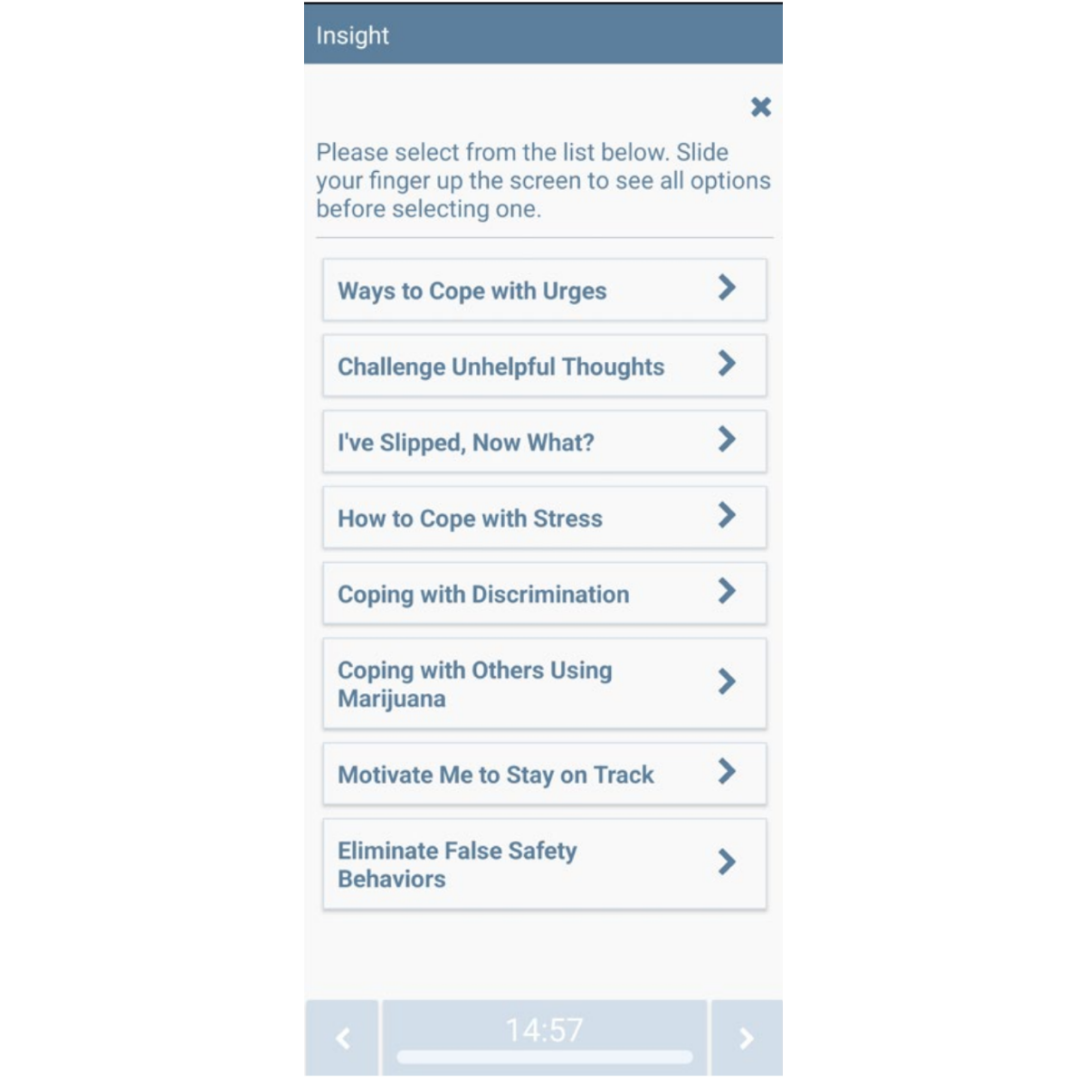
Coping toolkit CT-MICART feature. CT-MICART: Culturally Tailored-Mobile Integrated Cannabis and Anxiety Reduction Treatment.

**Figure 4:**
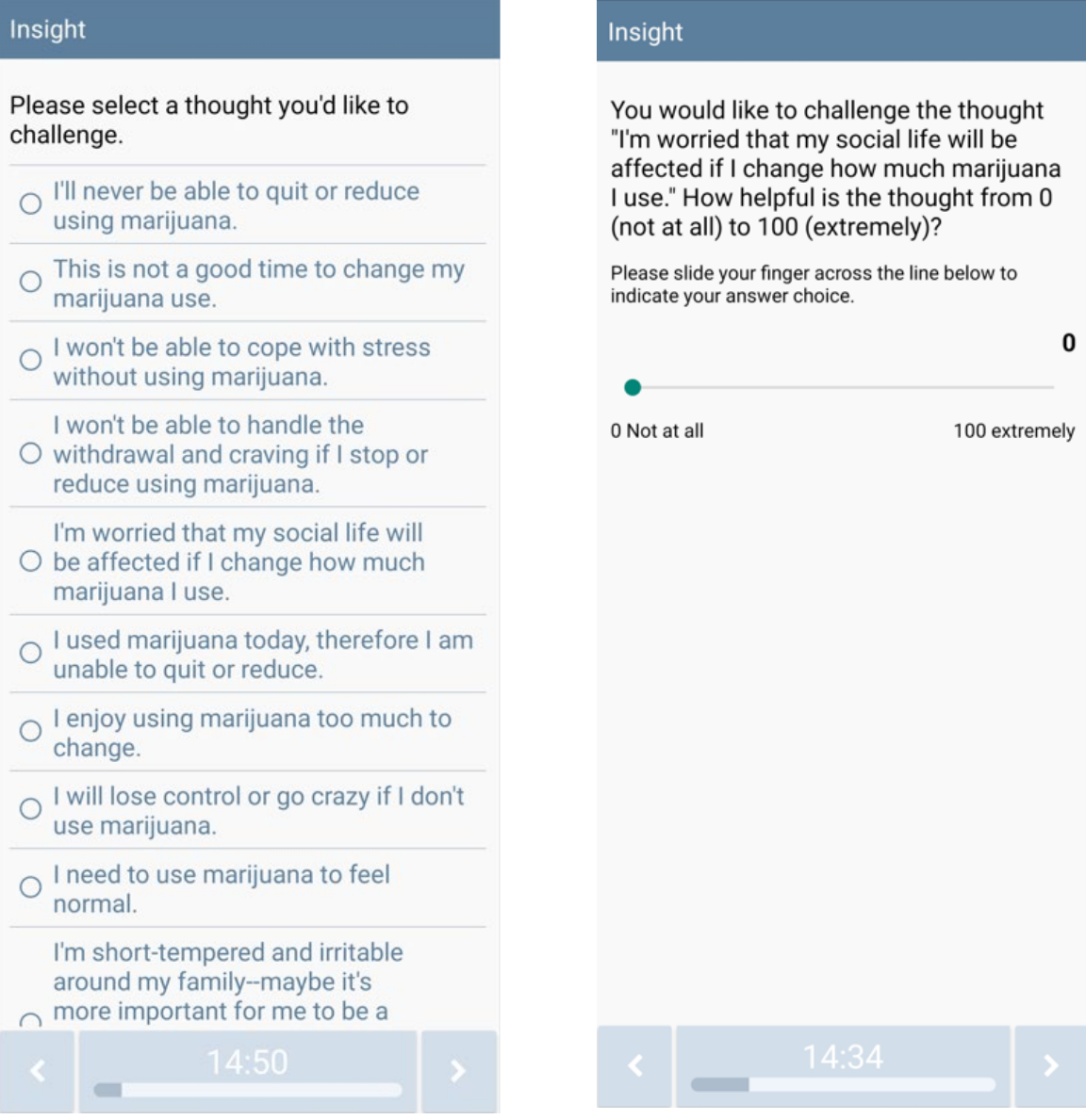
Challenging unhelpful thoughts CT-MICART feature. CT-MICART: Culturally Tailored-Mobile Integrated Cannabis and Anxiety Reduction Treatment.

**Figure 5:**
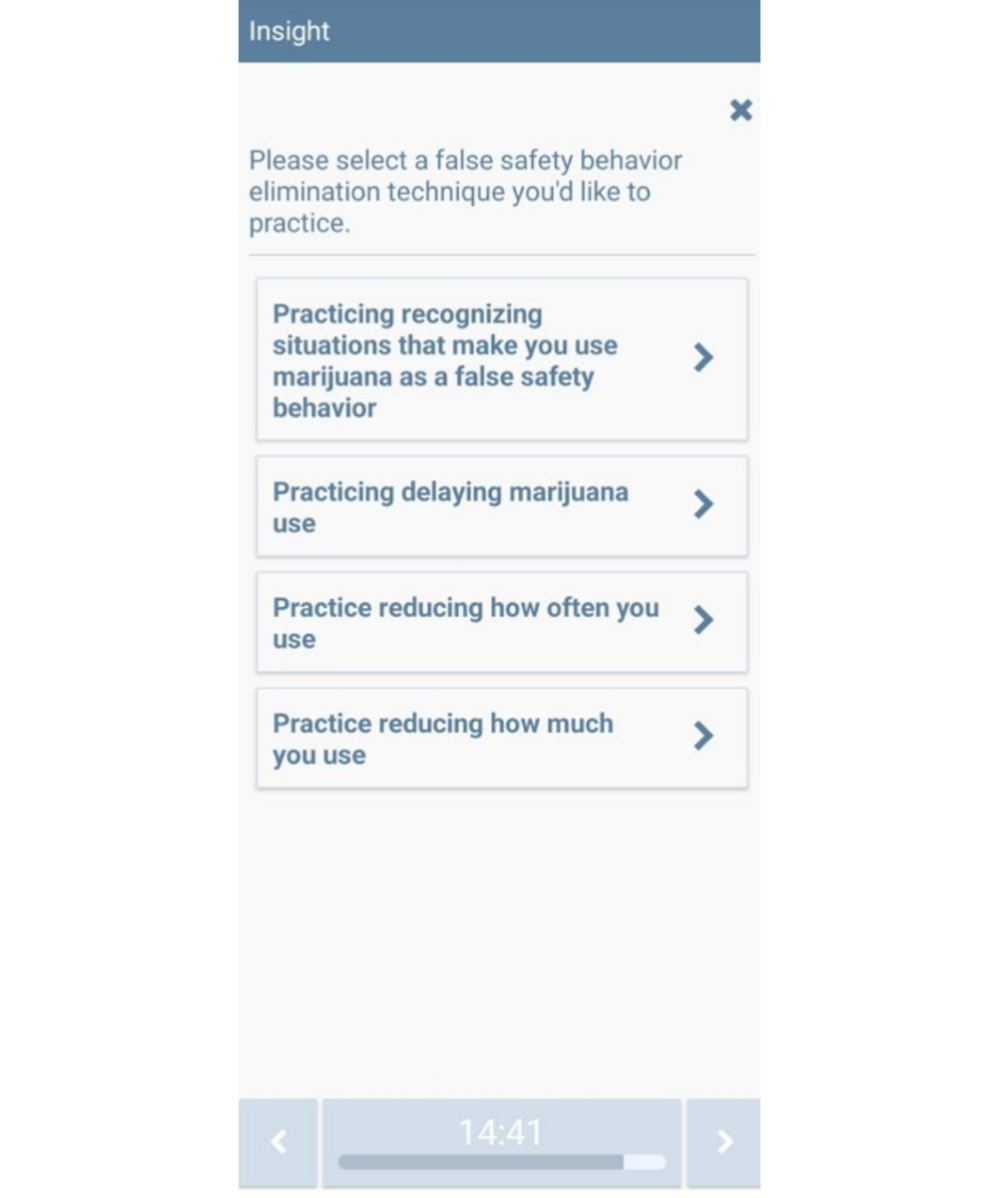
False safety behavior elimination CT-MICART feature. CT-MICART: Culturally Tailored-Mobile Integrated Cannabis and Anxiety Reduction Treatment.

**Figure 6:**
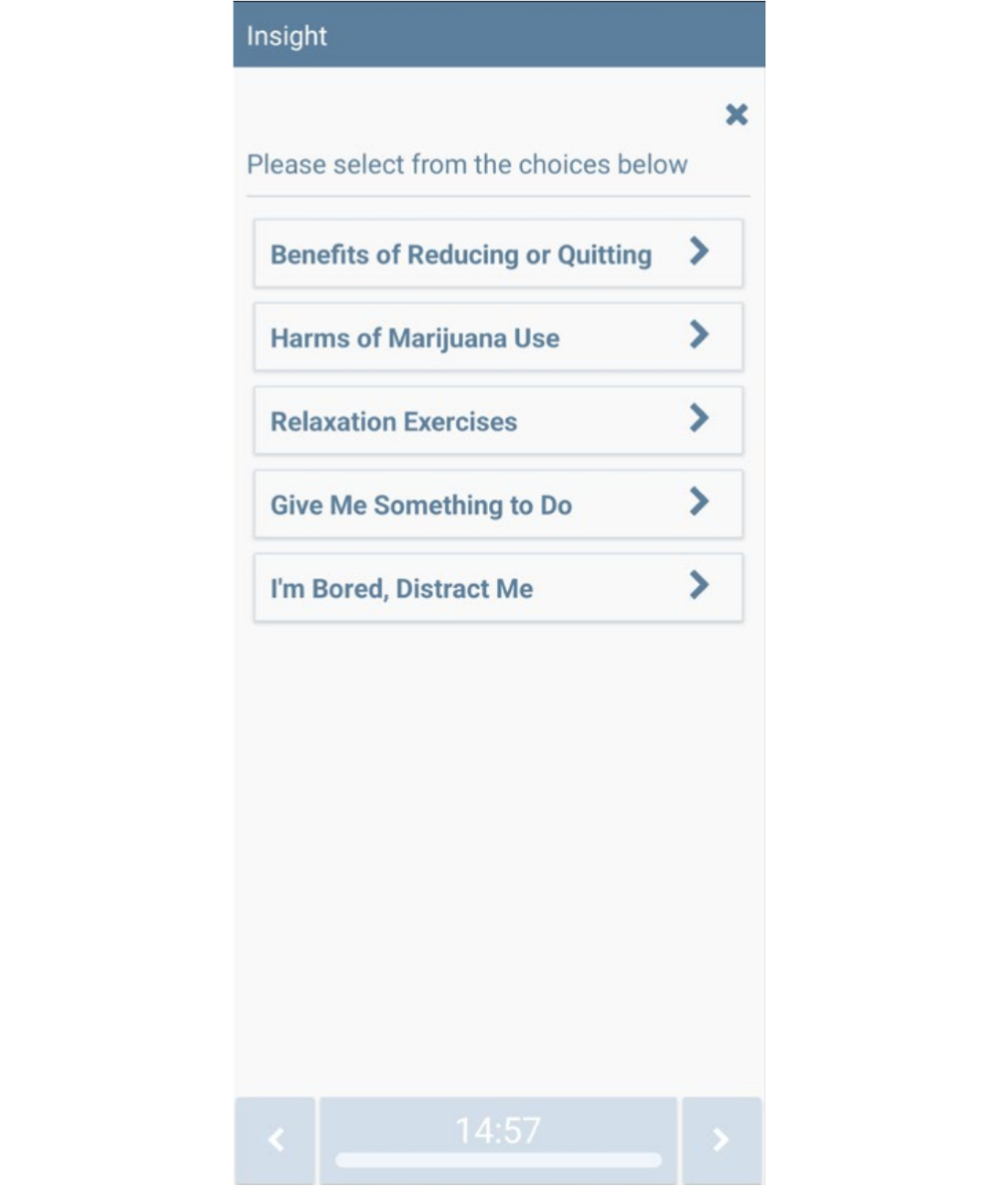
Help me reach my Goal CT-MICART feature. CT-MICART: Culturally Tailored-Mobile Integrated Cannabis and Anxiety Reduction Treatment.

Hundreds of unique messages were developed for this study to address various use risk triggers and to reduce repetition. Messages are consistent with MET-CBT approaches (including ICART) [31] and address identified cannabis use triggers. On-demand content is available through 2 buttons on the app home screen. First, the Coping Toolkit feature contains a menu of resources that become available once this button is pressed (see Figure 3), including Ways to Cope with Urges; Challenge Unhelpful Thoughts (see Figure 4 for a list of automatic thoughts that can be challenged via the app); I’ve Slipped, Now What?; How to Cope with Stress; Coping with Discrimination; Coping with others Using Marijuana; Motivate Me to Stay on Track; and Eliminate False Safety Behaviors (see Figure 5 for menu options). Second, the Help me Reach my Goal feature contains a menu of resources that also become available once this button is pressed (see Figure 6), including Benefits of Reducing or Quitting; Harms of Marijuana Use; Relaxation Exercises; Give Me Something to Do; and I’m Bored, Distract Me. Finally, all participants are instructed to click the “Record Stress or Urge,” “I’m About to Use,” and “I Just Used” buttons (see Figure 2) when appropriate. CT-MICART+EMA participants receive a tailored intervention message at the completion of each participant initiated EMAs.

#### Control Condition (EMAs Only)

Participants randomized to EMA only condition receive the baseline, follow-up, qualitative interview, 3 prompted daily EMAs for 6 weeks, and they have access to the participant-initiated “Record Stress or Urge,” “I’m About to Use,” and “I Just Used” buttons. However, they do not receive tailored messages or access to the CT-MICART intervention content.

### Assessments

#### Overview

See Table 1 for a schedule of data collection and measures list. All measures have been used among samples of Black adults.

**Table 1:** Measures and schedule for data collection.

Measure name	Screener	Baseline	6-week follow-up	EMA^a^ items
Demographics or background information	✓			Cannabis use assessment (outcomes)
Motivation to quit cannabis (eligibility)	✓			Alcohol consumption
REALM-SF^b^ (eligibility)	✓			Motivation regarding cannabis use
CUDIT-R^c^ (eligibility)	✓		✓	Watch treatment videos (CT-MICART^d^ condition only)
Legal mandate status (eligibility)	✓			Cannabis use goal assessment (CT-MICART condition only)
Report of cannabis use to manage anxiety or stress in the past month (eligibility)	✓			Negative affect
Assessment of ongoing substance use treatment or anxiety and depression (eligibility)	✓			Confidence in reducing or quitting
Safety Aid Scale (outcome)		✓	✓	Urges or cravings
Qualitative interview (outcome)			✓	Social support and daily interactions
System Usability Scale (outcome)			✓	Coping motives for cannabis and consequences or benefits of using
Credibility or Expectancy questionnaire (outcome)		✓	✓	False safety behavior elimination engagement
Marijuana Motives Measure (outcome)		✓	✓	N/A^e^

^a^EMA: ecological momentary assessment.

^b^REALM-SF: Rapid Estimate of Adult Literacy in Medicine-Short Form.

^c^CUDIT-R: Cannabis Use Disorder Identification Test.

^d^CT-MICART: Culturally Tailored-Mobile Integrated Cannabis and Anxiety Reduction Treatment.

^e^N/A: not applicable.

#### Study Screening and Demographics Questionnaire

All participants are asked to answer questions related demographic characteristics during the Qualtrics screener (eg, race, ethnicity, age, sex, education, and marital status). In addition, participants answer questions related to previous substance use, discrimination experiences, acculturation, anxiety, depression, motivation to quit or reduce cannabis use (ie, “On a scale from 1 to 10 with 1 being ‘not at all motivated’ and 10 being ‘extremely motivated’ how motivated are you to reduce your marijuana use in the next month?”), legal status to engage in substance use treatment, use of cannabis in the past month to cope with anxiety or stress, and ongoing treatment of substance use, anxiety, or depression.

#### REALM-SF Assessment

The REALM-SF is designed to determine if participants have at least a sixth-grade literacy level through a 7-item assessment taken with the help of a research assistant [37]. The REALM-SF is used in this study by the research staff to determine if participants can read the EMA items and intervention content.

#### CUDIT-R Assessment

The CUDIT-R is an 8-item self-report measure designed to identify the likelihood of CUD [35]. The items are rated on a 5-point Likert-type scale where higher scores indicate a higher likelihood of CUD. Scores of 12 or greater indicate probable CUD and it is used as eligibility criteria for this study [35].

#### Safety Aid Scale

The Safety Aid Scale (SAS) is an 80-item measure that is designed to assess FSBs in participants [24]. Examples of items and FSBs include “Avoid being far from home” and “Fiddling with an object (eg, pen).” The items are rated based on how frequently participants endorse the behavior on a scale from 0 (never or rarely) to 4 (almost always). Items are summed to form a total score of FSB use. This measure has demonstrated excellent internal consistency among Black adults [21]. For this study, item reduction analysis was used to reduce the number of items to 22 [39].

#### Marijuana Motives Measure

The Marijuana Motives Measure (MMM) is a 25-item questionnaire that assesses 5 motives for cannabis use including enhancement, coping, social, conformity, and expansion regarding motives for, frequency of, and problems associated with cannabis use [40]. Items are rated on a 5-point Likert-type scale where 1=almost never or never, 2=some of the time, 3=half of the time, 4=most of the time, and 5=almost always or always.

#### System Usability Scale

The System Usability Scale (SUS) is a 10-item questionnaire that is used in this study to evaluate participants’ perceived usability of the app [41]. The SUS includes items related to app engagement, frequency of use, complexity of the app, and thoughts about using the app.

#### Credibility or Expectancy Questionnaire

The Credibility or Expectancy Questionnaire (CEQ) is a 6-item measure that assesses treatment expectancy and rationale credibility [42]. It derives 2 predicted factors including cognitively based credibility and affectively based expectancy. In this study, it is used to gauge participants’ thoughts on the credibility of the intervention and assessment content assigned to them (CT-MICART+EMA or EMA only), and their expectations regarding the intervention (ie, “How successful do you think this intervention will be in helping you quit or reduce marijuana?”).

#### Daily EMAs

Throughout the 6-week intervention period, participants complete 3 daily EMAs occurring at specific times (one 30 minutes after self-selected wake time, one 60 minutes before the self-selected sleep time, and one at 12:15 PM). Items include questions about cannabis use, duration of being high, urges or cravings, motivation, and confidence to reduce or quit cannabis, negative affect, social support, and other substance use. Each EMA also gauges the CT-MICART+EMA participants’ cannabis goal for the day (ie, wanting to avoid using cannabis today, reduce use today, or no cannabis use goal today). All participants are also instructed to access the app’s on-demand assessment features, including the “Record Stress or Urge,” “I’m About to Use,” and “I just used” buttons, when they experience increased stress or an urge to use cannabis as well as anytime they think they might use or after actual cannabis use. Each time participants click these buttons, they are asked to complete a brief set of questions pertaining to their current situation.

#### Qualitative Interview

After completing the 6-week follow-up assessment in the app, study staff are notified via an encrypted email to contact the participant to complete the qualitative interview phone call. Participants are asked to assess their satisfaction with their assigned app content and ways it could be improved via a semistructured interview. This 30-minute interview assesses participant engagement with the app, and specific thoughts about app features including EMA items, intervention content, and why they did or did not use certain features.

### Data Analysis

#### General Overview

Given this pilot study is likely underpowered to detect statistical significance, conclusions will primarily be based on effect sizes and associated Cis, which will be used to guide future, larger, and fully powered trials of comparative efficacy. Prior to data analysis, we will assess the equivalence of groups on key baseline variables. Variables on which the groups differ will be used as covariates in the final analyses.

#### Hypothesis Testing

##### Overview

We will examine treatment effects on cannabis use (derived from EMA data) using multilevel models. We will focus on two cannabis use outcomes: (1) use on a given day and (2) frequency of use within a given day. We will account for baseline covariates and time since the start of the intervention. We will also use appropriate random effects and residual constraints. For coping motives for cannabis and FSB engagement, we will conduct a linear regression analysis wherein we will regress treatment condition on each outcome assessed at the 6-week follow-up while adjusting for baseline scores of the specific outcome and baseline covariates. Triangulation mixed methods quantitative or qualitative data analysis [43] will be used to evaluate quantitative and qualitative data.

##### Quantitative Data

The app’s feasibility and use will be examined by quantifying the use of CT-MICART features (eg, the number of assigned videos that are watched) and by evaluating participant opinions about the helpfulness of CT-MICART features (eg, treatment videos, automated treatment messages that follow EMAs, and exercises). Quantitative data analysis will focus on (1) behavioral markers of engagement with the app; (2) overall evaluations of the app and evaluations of each app feature, including usefulness or helpfulness and likelihood to recommend the app to a friend; and (3) data from the CEQ and SUS [44-46]. Data will be compared across conditions.

##### Qualitative Data

The week 6 qualitative interviews will prompt information on what participants liked about CT-MICART, how it could be improved, and what barriers currently limit app engagement. Individual interviews will be transcribed following the completion of participant treatment and then reviewed by the research team to ensure data quality. Transcribed interviews will be coded using NVivo (version 12; Lumivero), and decisions regarding the appropriateness of suggested changes to potential future versions of CT-MICART will be evaluated using a team-based approach. Consistent with the systematic and reflexive interviewing and reporting method [47], this approach will help to systematically organize collected qualitative data and thus guide improvements to CT-MICART. Moreover, content analysis will be used to analyze collected qualitative data [48]. Participant responses will be integrated with quantitative usage data to identify inconsistencies in the participant’s perceptions and actual engagement with the app [49,50].

#### Missing Data

Some participant attrition is anticipated to happen during this study. We will assume a missing-at-random mechanism if missing data occurs. This will allow us to increase statistical power and provide more accurate estimates of model parameters and standard errors, as they are the recommended intent-to-treat approach for clinical trials. We will compare this approach and the intent-to-treat approach as a sensitivity check of the influence of missing data on the statistical conclusion.

## Results

This study’s enrollment started in June 2023 and ended in November 2023. The estimated study completion date is March 2024.

## Discussion

### Principal Findings

Black adults evince significant cannabis-related health disparities compared with non-Black populations [13,15,51]. Thus, the primary goal of this study is to develop and pilot-test a culturally adapted mobile app for Black adults with probable CUD to help mitigate these disparities. We have culturally tailored this mobile intervention following the Cultural Accommodation Model [38], incorporating knowledge from the current research team, published literature, expert opinion, and feedback from the CRAB. As a next step, we seek to obtain data on the initial efficacy and qualitative evaluation of the app with the target population (ie, Black adults with probable CUD). To our knowledge, this is the first culturally tailored mHealth intervention to integrate FSB and CBT for CUD for Black adults with a probable CUD. We hypothesize that the culturally tailored mobile app CT-MICART will lead to reduced cannabis use and related problems and reduce the use of FSBs.

Though extant literature has highlighted existing mHealth work for cannabis use and CUD [32], including mHealth interventions tailored to treat specific populations (ie, individuals with psychosis and comorbid substance use) [33,34], this study is the first to target specific psycho-sociocultural factors related to cannabis use and use-related problems among Black cannabis users through a mobile intervention. Should the CT-MICART app prove efficacious in reducing cannabis use, it will provide health care officials and researchers a unique opportunity to further refine and provide low-cost and easily accessible treatment for a historically underrepresented and underserved population. The success of the CT-MICART app would also provide the foundation to further refine and culturally inform better implementation of the app through qualitative interviews of all participants. Overall, the CT-MICART app and its development provide the potential to address the dearth of literature that exists for mobile health development and Black individuals who use cannabis.

### Limitations

This study has several limitations which warrant comment. First, this pilot randomized controlled trial (RCT) will only offer intervention content and collect data for 6 weeks. As such, future research should examine the efficacy of similar but longer-term interventions. Second, as the study will only enroll participants that are motivated to quit or reduce their cannabis use, future work should determine if participants who are not currently motivated to quit or reduce their cannabis use can benefit from this type of culturally tailored cannabis cessation or reduction app. Third, only Android smartphone users will be enrolled in this study due to the limitations of the Insight smartphone platform. Future studies will use the updated Insight platform that works on Apple and Android smartphones. Additionally, it is possible that the EMA-only condition will have potentially therapeutic benefits to participants due to self-monitoring of behaviors, although EMA studies on substance use have found such effects to be limited [52-54]. Thus, a waitlist control should be included in future study designs to isolate the effect of CT-MICART beyond the potential influence of behavior monitoring or tracking behavior. Finally, although the sample size will be adequate to achieve study aims or hypotheses, future fully powered studies will be needed to test intervention efficacy and effectiveness.

### Conclusions

This study will address a significant gap in the literature. Specifically, this pilot RCT will offer initial insights on the use of an mHealth intervention that is tailored for Black individuals who use cannabis. Smartphone interventions similar to CT-MICART have incredible potential to provide low-cost, scalable treatments to diverse populations. Moreover, the CT-MICART app has the potential to help Black individuals who use cannabis achieve and maintain higher rates of cessation and reduction and help narrow health disparities that have negatively impacted this underserved population.

## Abbreviations

CBT: cognitive behavioral therapy
CEQ: Credibility or Expectancy Questionnaire
CRAB: Community Research Advisory Board
CT-MICART: Culturally Tailored-Mobile Integrated Cannabis and Anxiety Reduction Treatment
CUD: cannabis use disorder
CUDIT-R: Cannabis Use Disorder Identification Test-Revised
EMA: ecological momentary assessment
FSB: false safety behavior
ICART: Integrated Cannabis and Anxiety Reduction Treatment
IRB: institutional review board
MET-CBT: motivation enhancement therapy combined with cognitive behavioral therapy
mHealth: mobile health
MMM: Marijuana Motives Measure
RCT: randomized controlled trial
REALM-SF: Rapid Estimate of Adult Literacy in Medicine-Short Form
SAS: Safety Aid Scale
SUS: System Usability Scale

## Data Availability

The data sets generated and analyzed during this study are not yet publicly available given that data analysis in currently in progress but will be available from the corresponding author on reasonable request.

## References

[1] Johnston LD, O’Malley PM, Bachman JG (2003). Monitoring the future: national results on adolescent drug use: overview of key findings. Focus.

[2] (2022). 2022 NSDUH annual national report. Substance Abuse and Mental Health Services Administration.

[3] (2023). Marijuana and hallucinogen use, binge drinking reached historic highs among adults 35 to 50. National Institute on Drug Abuse.

[4] Wu LT, Zhu H, Swartz MS (2016). Trends in cannabis use disorders among racial/ethnic population groups in the United States. Drug Alcohol Depend.

[5] Mantey DS, Onyinye ON, Montgomery L (2021). Prevalence and correlates of daily blunt use among U.S. African American, Hispanic, and White adults from 2014 to 2018. Psychol Addict Behav.

[6] Hasin DS, Saxon AJ, Malte C, Olfson M, Keyes KM, Gradus JL, Cerdá M, Maynard CC, Keyhani S, Martins SS, Fink DS, Livne O, Mannes Z, Wall MM (2022). Trends in cannabis use disorder diagnoses in the U.S. Veterans Health Administration, 2005-2019. Am J Psychiatry.

[7] Foster KT, Arterberry BJ, Iacono WG, McGue M, Hicks BM (2018). Psychosocial functioning among regular cannabis users with and without cannabis use disorder. Psychol Med.

[8] Masters MN, Haardörfer R, Windle M, Berg C (2018). Psychosocial and cessation-related differences between tobacco-marijuana co-users and single product users in a college student population. Addict Behav.

[9] McClure EA, Tomko RL, Salazar CA, Akbar SA, Squeglia LM, Herrmann E, Carpenter MJ, Peters EN (2019). Tobacco and cannabis co-use: drug substitution, quit interest, and cessation preferences. Exp Clin Psychopharmacol.

[10] Copersino ML, Boyd SJ, Tashkin DP, Huestis MA, Heishman SJ, Dermand JC, Simmons MS, Gorelick DA (2010). Sociodemographic characteristics of cannabis smokers and the experience of cannabis withdrawal. Am J Drug Alcohol Abuse.

[11] Warner TD (2016). Up in smoke: neighborhood contexts of marijuana use from adolescence through young adulthood. J Youth Adolesc.

[12] Kennedy BR, Mathis CC, Woods AK (2007). African Americans and their distrust of the health care system: healthcare for diverse populations. J Cult Divers.

[13] Montgomery L, Dixon S, Mantey DS (2022). Racial and ethnic differences in cannabis use and cannabis use disorder: implications for researchers. Curr Addict Rep.

[14] Budney AJ, Roffman R, Stephens RS, Walker D (2007). Marijuana dependence and its treatment. Addict Sci Clin Pract.

[15] Montgomery L, Robinson C, Seaman EL, Haeny AM (2017). A scoping review and meta-analysis of psychosocial and pharmacological treatments for cannabis and tobacco use among African Americans. Psychol Addict Behav.

[16] Martins SS, Segura LE, Levy NS, Mauro PM, Mauro CM, Philbin MM, Hasin DS (2021). Racial and ethnic differences in cannabis use following legalization in US States with medical cannabis laws. JAMA Netw Open.

[17] Solomon R (2020). Racism and its effect on cannabis research. Cannabis Cannabinoid Res.

[18] Buckner JD, Zvolensky MJ, Scherzer CR (2023). Microaggressions and cannabis-related problems among Black adults: the roles of negative affect and cannabis use motives. Cogn Ther Res.

[19] Buckner JD, Zvolensky MJ, Lewis EM (2020). Smoking and social anxiety: the role of false safety behaviors. Cogn Behav Ther.

[20] Schmidt NB, Buckner JD, Pusser A, Woolaway-Bickel K, Preston JL, Norr A (2012). Randomized controlled trial of false safety behavior elimination therapy: a unified cognitive behavioral treatment for anxiety psychopathology. Behav Ther.

[21] Buckner JD, Zvolensky MJ, Ferrie ML, Morris PE (2023). False safety behavior use among Black adults. Cogn Behav Ther.

[22] Lev-Ran S, Le Foll B, McKenzie K, George TP, Rehm J (2013). Cannabis use and cannabis use disorders among individuals with mental illness. Compr Psychiatry.

[23] van Ours JC, Williams J (2011). Cannabis use and mental health problems. J Applied Econometrics.

[24] Buckner JD, Zvolensky MJ, Ecker AH, Jeffries ER, Lemke AW, Dean KE, Businelle MS, Gallagher MW (2017). Anxiety and cannabis-related problem severity among dually diagnosed outpatients: the impact of false safety behaviors. Addict Behav.

[25] Buckner JD, Zvolensky MJ, Businelle MS, Gallagher MW (2018). Direct and indirect effects of false safety behaviors on cannabis use and related problems. Am J Addict.

[26] Buckner JD, Zvolensky MJ, Scherzer CR (2023). Alcohol-related problems among Black adults: the role of false safety behaviors. J Racial Ethn Health Disparities.

[27] Peraza N, Smit T, Garey L, Manning K, Buckner JD, Zvolensky MJ (2019). Distress tolerance and cessation-related cannabis processes: the role of cannabis use coping motives. Addict Behav.

[28] Buckner JD, Zvolensky MJ, Ecker AH, Schmidt NB, Lewis EM, Paulus DJ, Lopez-Gamundi P, Crapanzano KA, Bakhshaie J (2019). Integrated cognitive behavioral therapy for comorbid cannabis use and anxiety disorders: a pilot randomized controlled trial. Behav Res Ther.

[29] Buckner JD, Zvolensky MJ, Schmidt NB, Carroll KM, Schatschneider C, Crapanzano K (2014). Integrated cognitive behavioral therapy for cannabis use and anxiety disorders: rationale and development. Addict Behav.

[30] Buckner JD, Ecker AH, Beighley JS, Zvolensky MJ, Schmidt NB, Shah SM, Carroll KM (2016). Integrated cognitive behavioral therapy for comorbid cannabis use and anxiety disorders. Clin Case Stud.

[31] Buckner JD, Morris PE, Zvolensky MJ (2021). Integrated cognitive-behavioral therapy for comorbid cannabis use and anxiety disorders: the impact of severity of cannabis use. Exp Clin Psychopharmacol.

[32] Brezing CA, Levin FR (2022). Applications of technology in the assessment and treatment of cannabis use disorder. Front Psychiatry.

[33] Tatar O, Abdel-Baki A, Wittevrongel A, Lecomte T, Copeland J, Lachance-Touchette P, Coronado-Montoya S, Côté J, Crockford D, Dubreucq S, L'Heureux S, Ouellet-Plamondon C, Roy MA, Tibbo PG, Villeneuve M, Jutras-Aswad D (2022). Reducing cannabis use in young adults with psychosis using iCanChange, a mobile health app: protocol for a pilot randomized controlled trial (ReCAP-iCC). JMIR Res Protoc.

[34] Beckham JC, Adkisson KA, Hertzberg J, Kimbrel NA, Budney AJ, Stephens RS, Moore SD, Calhoun PS (2018). Mobile contingency management as an adjunctive treatment for co-morbid cannabis use disorder and cigarette smoking. Addict Behav.

[35] Adamson SJ, Kay-Lambkin FJ, Baker AL, Lewin TJ, Thornton L, Kelly BJ, Sellman JD (2010). An improved brief measure of cannabis misuse: the Cannabis Use Disorders Identification Test-Revised (CUDIT-R). Drug Alcohol Depend.

[36] Murphy PW, Davis TC, Long SW, Jackson RH, Decker BC (1993). Rapid Estimate of Adult Literacy in Medicine (REALM): a quick reading test for patients. J Read.

[37] Arozullah AM, Yarnold PR, Bennett CL, Soltysik RC, Wolf MS, Ferreira RM, Lee SYD, Costello S, Shakir A, Denwood C, Bryant FB, Davis T (2007). Development and validation of a short-form, rapid estimate of adult literacy in medicine. Med Care.

[38] Wieling E, Rastogi M (2005). Voices of Color: First-Person Accounts of Ethnic Minority Therapists.

[39] Jin X, Liu GG, Gerstein HC, Levine MAH, Steeves K, Guan H, Li H, Xie F (2018). Item reduction and validation of the Chinese version of Diabetes Quality-of-Life measure (DQOL). Health Qual Life Outcomes.

[40] Simons J, Correia CJ, Carey KB, Borsari BE (1998). Validating a five-factor marijuana motives measure: relations with use, problems, and alcohol motives. J Couns Psychol.

[41] Brooke J, Thomas B, Weerdmeester BA, McClelland IL, Jordan PW (1996). SUS: a "quick and dirty' usability scale. Usability Evaluation In Industry.

[42] Devilly GJ, Borkovec TD (2000). Psychometric properties of the credibility/expectancy questionnaire. J Behav Ther Exp Psychiatry.

[43] Wilson V (2014). Research methods: triangulation. Evid Based Libr Inf Pract.

[44] Rooke SE, Gates PJ, Norberg MM, Copeland J (2014). Applying technology to the treatment of cannabis use disorder: comparing telephone versus internet delivery using data from two completed trials. J Subst Abuse Treat.

[45] Hindson J, Hanstock T, Dunlop A, Kay-Lambkin F (2020). Internet-delivered tobacco treatment for people using cannabis: a randomized trial in two Australian cannabis clinics. JMIR Form Res.

[46] Macatee RJ, Albanese BJ, Okey SA, Afshar K, Carr M, Rosenthal MZ, Schmidt NB, Cougle JR (2021). Impact of a computerized intervention for high distress intolerance on cannabis use outcomes: a randomized controlled trial. J Subst Abuse Treat.

[47] Loubere N (2017). Questioning transcription: the case for the Systematic and Reflexive Interviewing and Reporting (SRIR) method. Forum Qual Sozialforschung.

[48] Bengtsson M (2016). How to plan and perform a qualitative study using content analysis. NursingPlus Open.

[49] Creswell JW, Clark VLP (2017). Designing and Conducting Mixed Methods Research.

[50] Cheney MK, Gowin M (2020). Using Legal Documents for Public Health Research: An Innovative Application of Qualitative Methodology to Document the Experiences of Transgender Asylum Seekers.

[51] Lanaway D, Burlew AK (2021). The influence of distressed coping on the relationship between perceived racial discrimination and cannabis use among Black college students. J Psychoactive Drugs.

[52] Litt MD, Cooney NL, Morse P (1998). Ecological Momentary Assessment (EMA) with treated alcoholics: methodological problems and potential solutions. Health Psychol.

[53] Hufford MR, Shields AL, Shiffman S, Paty J, Balabanis M (2002). Reactivity to ecological momentary assessment: an example using undergraduate problem drinkers. Psychol Addict Behav.

[54] Simpson TL, Kivlahan DR, Bush KR, McFall ME (2005). Telephone self-monitoring among alcohol use disorder patients in early recovery: a randomized study of feasibility and measurement reactivity. Drug Alcohol Depend.

